# Prediction of preload dependency using phenylephrine-induced peripheral perfusion index during general anaesthesia: a prospective observational study

**DOI:** 10.1186/s12871-024-02478-w

**Published:** 2024-03-02

**Authors:** Yusuke Iizuka, Koichi Yoshinaga, Shizuka Amitani, Seiya Nishiyama, Kentaro Fukano, Keika Miyazawa, Asuka Kitajima, Ikumi Sawada, Yuji Otsuka, Masamitsu Sanui

**Affiliations:** 1 Department of Anesthesiology and Critical Care Medicine, Jichi Medical University Saitama Medical Center, 1-847 Amanuma, Omiya-ku, Saitama City, Saitama 330-8503 Japan; 2 Department of Anesthesiology and Critical Care Medicine, Jichi Medical University, 3311- 1 Yakushiji, Shimotsuke, Tochigi, 329-0498 Japan; 3 Division of Critical Care, Department of Anesthesiology and Critical Care Medicine, Jichi Medical University, 3311-1 Yakushiji, Shimotsuke, Tochigi, 329-0498 Japan

**Keywords:** Phenylephrine, Fluid responsiveness, Peripheral perfusion index, Stroke volume

## Abstract

**Background:**

Tracking preload dependency non-invasively to maintain adequate tissue perfusion in the perioperative period can be challenging.The effect of phenylephrine on stroke volume is dependent upon preload. Changes in stroke volume induced by phenylephrine administration can be used to predict preload dependency. The change in the peripheral perfusion index derived from photoplethysmography signals reportedly corresponds with changes in stroke volume in situations such as body position changes in the operating room. Thus, the peripheral perfusion index can be used as a non-invasive potential alternative to stroke volume to predict preload dependency. Herein, we aimed to determine whether changes in perfusion index induced by the administration of phenylephrine could be used to predict preload dependency.

**Methods:**

We conducted a prospective single-centre observational study. The haemodynamic parameters and perfusion index were recorded before and 1 and 2 min after administering 0.1 mg of phenylephrine during post-induction hypotension in patients scheduled to undergo surgery. Preload dependency was defined as a stroke volume variation of ≥ 12% before phenylephrine administration at a mean arterial pressure of < 65 mmHg. Patients were divided into four groups according to total peripheral resistance and preload dependency.

**Results:**

Forty-two patients were included in this study. The stroke volume in patients with preload dependency (*n* = 23) increased after phenylephrine administration. However, phenylephrine administration did not impact the stroke volume in patients without preload dependency (*n* = 19). The perfusion index decreased regardless of preload dependency. The changes in the perfusion index after phenylephrine administration exhibited low accuracy for predicting preload dependency. Based on subgroup analysis, patients with high total peripheral resistance tended to exhibit increased stroke volume following phenylephrine administration, which was particularly prominent in patients with high total peripheral resistance and preload dependency.

**Conclusion:**

The findings of the current study revealed that changes in the perfusion index induced by administering 0.1 mg of phenylephrine could not predict preload dependency. This may be attributed to the different phenylephrine-induced stroke volume patterns observed in patients according to the degree of total peripheral resistance and preload dependency.

**Trial registration:**

University Hospital Medical Information Network (UMIN000049994 on 9/01/2023).

**Supplementary Information:**

The online version contains supplementary material available at 10.1186/s12871-024-02478-w.

## Background

Preload dependency or fluid responsiveness guides the intraoperative administration of fluids and aids in achieving adequate tissue perfusion [1]. Stroke volume variation (SVV) and pulse pressure variation (PPV) are widely used to predict preload dependency during the intraoperative period [2, 3]. However, invasive devices, such as arterial catheters, are inevitably used to estimate the changes in stroke volume (SV) to measure these parameters. Given that arterial catheter insertion is not possible for all patients undergoing surgery, an alternative non-invasive method is needed to assess preload dependency. Currently, several non-invasive devices that measure SV are available, including the ClearSight system (Edwards Lifesciences, Irvine, CA, USA), the CNAP monitor (CNSsytems Medizintechnik AG, Graz, Austria), and the Nicom Reliant system (Cheetah Medical, Vancouver, WA, USA). However, these devices require dedicated equipment and are costly.

Peripheral perfusion index (PI) is the ratio of the pulsatile and non-pulsatile components of the arterial waveform derived from the photoplethysmography signal and is influenced by various factors, such as venous return, cardiac output, arterial stiffness, vascular tone, body position, peripheral temperature and peripheral compression [4]. Notably, change in SV is an important factor that affects the value of PI and vascular tone. Several studies have shown that changes in PI correspond with changes in SV following body position changes (e.g. during head-up tilt or passive leg raising test) in the operating room and intensive care unit [5, 6]. Therefore, PI may be correlated with SV under stable vascular tone conditions.

Pleth variability index (PVI), the variation of PI in the respiratory cycle, has been reported to predict preload dependency with high accuracy [7] and may be an alternative to SVV and PPV. Although PVI is a valuable non-invasive tool to predict preload dependency, it can only be monitored using Masimo technology (Masimo Corp, Irvine, CA, USA). Thus, using PI to predict preload dependency can enhance the value of research for anaesthesiologists owing to its wider applicability on various monitoring systems other than Masimo.

In addition to fluid management, appropriate hypotension treatment plays an important role in maintaining adequate tissue perfusion during general anaesthesia. Intraoperative hypotension can be induced by vasodilation owing to the excessive depth of anaesthesia, neuraxial blockade, arrhythmias, myocardial ischemia and hypovolemia. Although treatment should be optimised according to each cause, fluid and vasopressor drugs are used simultaneously in most cases, given that the use of only vasopressor can lead to a decrease in SV due to increased afterload, especially in patients with hypovolemia. However, non-invasive differentiation of patients experiencing intraoperative hypotension, with and without hypovolemia, can facilitate hypotension management and prioritise treatment options, such as fluids or vasopressors.

Phenylephrine, a vasopressor drug typically used during general anaesthesia, is a direct-acting, predominantly alpha_1_-adrenergic receptor agonist capable of influencing the SV. Elevated SV is attributed to venoconstriction and conversion of unstressed to stressed volume (increasing preload), while the reduction in SV occurs due to the restriction of venous return (increased venous resistance) and increasing afterload [8].

While few studies have investigated the association between vasopressor administration (both phenylephrine and noradrenaline) and preload dependency, their results indicate that phenylephrine-induced changes in SV differed with preload dependency. For example, Rebet et al. demonstrated that the effect of phenylephrine administration (0.05–0.15 mg) on SV differed between groups with and without preload dependency during general anaesthesia, with reduced SV observed in patients without preload dependency but not in those with preload dependency [9]. The different patterns of SV changes following phenylephrine administration may be attributed to changes in venous return and afterload. Thus, the increase in venous return offsets the decrease in SV due to increased afterload in the preload-dependent groups. Conversely, the decrease in SV due to increased afterload can overcome the increase in venous return in the non-preload-dependent groups using two simple models: the venous return and cardiac function curves. Maas et al. investigated the effect of norepinephrine administration on the haemodynamic parameters in postoperative cardiac surgery patients [10]. Although norepinephrine exerts a considerable beta-adrenergic effect, results demonstrating that hemodynamic changes differed by preload dependency following administration have been reported. The pre-administration SVV was 14.4 ± 4.2% in the increased cardiac output (CO) group and 9.1 ± 2.4% in the decreased CO group. In the increased CO group, the increase in CO was attributed to the increase in SV, owing to the lack of increase in heart rate (HR). In the decreased CO group, the decrease in CO was caused by the decrease in HR, given that SV was unaltered. However, the different response patterns in both SV and CO after norepinephrine administration were attributed to the balance between the increase in venous return and the decrease in SV by increasing the afterload and altering the resistance of venous return. Moreover, SVV > 8.7% was found to be associated with increased CO following norepinephrine administration. Accordingly, the findings of the above-discussed studies demonstrate that the change in SV or CO after vasopressor administration (phenylephrine or norepinephrine) may depend on preload dependency (i.e., the SVV value prior to administration).

Therefore, changes in SV in response to phenylephrine may be a novel predictor of preload dependency, and PI may be a valid non-invasive alternative parameter to SV. However, to the best of our knowledge, no study has evaluated whether phenylephrine-induced changes in PI can predict preload dependency.

In the current study, we aimed to determine whether the pattern of PI change following phenylephrine administration could predict preload dependency during hypotension under general anaesthesia. We hypothesized that SV and PI would increase or remain unchanged following phenylephrine in patients with preload dependency and decrease in patients without dependency. Moreover, we determined the optimal timing to assess PI after phenylephrine administration based on haemodynamic parameters.

## Methods

### Ethics

This single-centre prospective study was approved by the Research Ethics Committee of Jichi Medical University Saitama Medical Centre Saitama, Japan (Chairperson Prof. Shinichi Kako) on 4/01/2023 (approval number: S22-092). The requirement for obtaining written informed consent was waived by the Research Ethics Committee of Jichi Medical University Saitama Medical Center owing to the non-invasive nature of the study. This study was conducted in accordance with the Declaration of Helsinki and the Strengthening the Reporting of Observational Studies in Epidemiology statement for observational studies [11]. The study was registered in the University Hospital Medical Information Network (registration number: UMIN000049994 on 9/01/2023).

### Patients

Patients older than 18 years who were scheduled for surgery and who underwent radial artery catheter placement and cardiac output monitoring between January and April 2023 were included in this study. Exclusion criteria were severe preoperative lung disease (Chronic Obstructive Pulmonary Disease clinical stage of Global Initiative for Chronic Obstructive Lung Disease [GOLD] 3 or 4 or a history of lung resection), left ventricular ejection fraction of ≤ 40%, atrial fibrillation, right heart failure (suspected pulmonary hypertension or elevated central venous pressure), obesity (body mass index ≥ 35 kg/m^2^), emaciation (body mass index < 15 kg/m^2^), peripheral artery disease, haemodialysis, hyperthyroidism, bradycardia (HR ≤ 50 bpm/min), and brachial plexus block administration.

### Study setting

All patients were admitted to the hospital the day prior to surgery or earlier. The patients were instructed to fast in the morning on the day of surgery but were allowed to drink clear fluids for up to 2 h before the start of surgery. Standard monitoring, including three- or five-lead electrocardiography, non-invasive blood pressure monitoring, and pulse oximetry, was initiated in the operating room. Propofol (1 − 2 mg/kg bolus or started 3 − 5 μg/mL on targeted controlled infusion [TCI]), remifentanil (0.2 − 0.5 μg/kg/min), and rocuronium (0.6 − 1.0 mg/kg) were used for the induction of anaesthesia and tracheal intubation. Anaesthesia was maintained using remifentanil (0.2 − 0.5 μg/kg/min) combined with propofol (2 − 3 μg/mL on TCI), remimazolam (0.5 − 1.0 mg/kg/h), sevoflurane (1 − 1.5%), or desflurane (3 − 5%). After induction of anaesthesia, the rate of fluid infusion was set to 2–3 mL/kg/h. The patients were mechanically ventilated using the volume-controlled mode, with the tidal volume set to 8 mL/predicted body weight (PBW). PBW was calculated using the following formula [12]:

$${\rm{Male}}:\,{\rm{50}}\,{\rm{ + }}\,\left( {{\rm{0}}.{\rm{91}}\, \times \,\left[ {{\rm{height}}\,{\rm{in}}\,{\rm{cm}} - {\rm{152}}.{\rm{4}}} \right]} \right)$$

$${\rm{Female}}:\,45.5\,{\rm{ + }}\,\left( {{\rm{0}}.{\rm{91}}\, \times \,\left[ {{\rm{height}}\,{\rm{in}}\,{\rm{cm}} - {\rm{152}}.{\rm{4}}} \right]} \right)$$

The ventilation frequency was adjusted to maintain the end-tidal carbon dioxide concentration between 34 and 38 mmHg. A positive end-expiratory pressure of 5 cm H_2_O was applied.

### Measurements

In addition to standard anaesthesia monitoring, a radial arterial catheter was inserted in all patients and connected to a Flotrac™ sensor (version 4.00. Edwards, Irvine, CA, USA) placed at the level of the phlebostatic axis. SV and SVV were monitored using a HemoSphere Advanced Monitoring Platform (Edwards, Irvine, CA, USA). PI was measured using Radical-7 (Masimo Corp., Irvine, CA, USA) at the middle finger of the contralateral arm, into which the arterial line was inserted to avoid the influence of arterial catheterisation on the digital perfusion [13, 14]. The mode of display of PI was set to the “long-term” mode, which displays the averaged PI value over 30 s.

The hemodynamic parameters (HR, systolic/diastolic/mean arterial pressure [SAP/DAP/MAP, respectively], pulse pressure [PP], CO, cardiac index [CI], SV, stroke volume index [SVI], SVV, PPV, PI, PVI, total peripheral resistance [TPR] and Dynamic arterial elastance [Eadyn]), respiratory setting, bispectral index (BIS), and finger temperature were recorded at post-induction hypotension (MAP was < 65 mmHg). TPR was calculated as MAP/CO × 80. Eadyn was calculated as PPV/SVV. The haemodynamic parameters were also collected at 1 and 2 min after 0.1 mg of phenylephrine administration. All measurements were performed between the induction of general anaesthesia and the skin incision.

### Statistical analysis

Patients with preload dependency were defined as having an SVV of ≥ 12% according to a meta-analysis that investigated the ability of SVV to predict preload dependency (an area under the receiver operating characteristic [ROC]: 0.84, mean threshold value: 11.6 ± 1.9%) [15]. Forty-two patients were required to demonstrate that the changes in PI induced by phenylephrine administration can predict preload dependency with an ROC curve of ≥ 0.75 (type I error of 5% and type II error of 20%). The Shapiro–Wilk test was used to assess the normality of continuous variables. Normally distributed variables are expressed as mean ± standard deviation [SD], whereas non-normally distributed variables are expressed as medians (interquartile range [IQR]). Where appropriate, the two-sample t-test and Mann–Whitney U test were used to compare the continuous variables between the groups. Categorical variables are expressed as numbers. Where appropriate, Fisher’s exact or chi-square tests were used to compare the categorical variables. Repeated measures analysis of variance was used to analyse the changes in the haemodynamic variables induced by phenylephrine administration within the groups (preload-dependent and preload-independent groups). The ROC curves were constructed to determine the ability of PI and other hemodynamic parameters to detect preload dependency, maximising sensitivity and specificity using Youden’s index.

The relationship between the change in SV due to phenylephrine administration and TPR, representing the afterload at post-induction hypotension, was evaluated to clarify the effect of phenylephrine on SV. Subgroup analysis was also performed on four subgroups: Group 1, high TPR + preload dependency; Group 2, Low TPR + preload dependency; Group 3, High TPR + preload independency; and Group 4, Low TPR + preload independency. The optimal cut-off value determined by ROC curves assessing the ability of TPR to detect SV increase established that a rate of change in SV ˃1.0 between post-induction hypotension and 2 min after phenylephrine could serve as the threshold for discriminating high or low TPR (The TPR threshold was determined as 1179 dyne-sec/cm^5^ as seen in results).

Pearson’s correlation coefficient (r) was used to evaluate the relationship between two values, and a regression equation was derived using the least squares method. Statistical significance was set at *P* < 0.05.

All statistical analyses were performed using EZR (Saitama Medical Centre, Jichi Medical University, Saitama, Japan), a graphical user interface for R (version 3.6.3, R Foundation for Statistical Computing, Vienna, Austria). EZR is a modified version of the R commander (version 2.6-2) designed to add statistical functions frequently used in biostatistics [16]. GraphPad Prism (version. 10.1.1, GraphPad Software LLC, Boston, MA, USA) was used for data visualization.

## Results

**Fig. 1:**
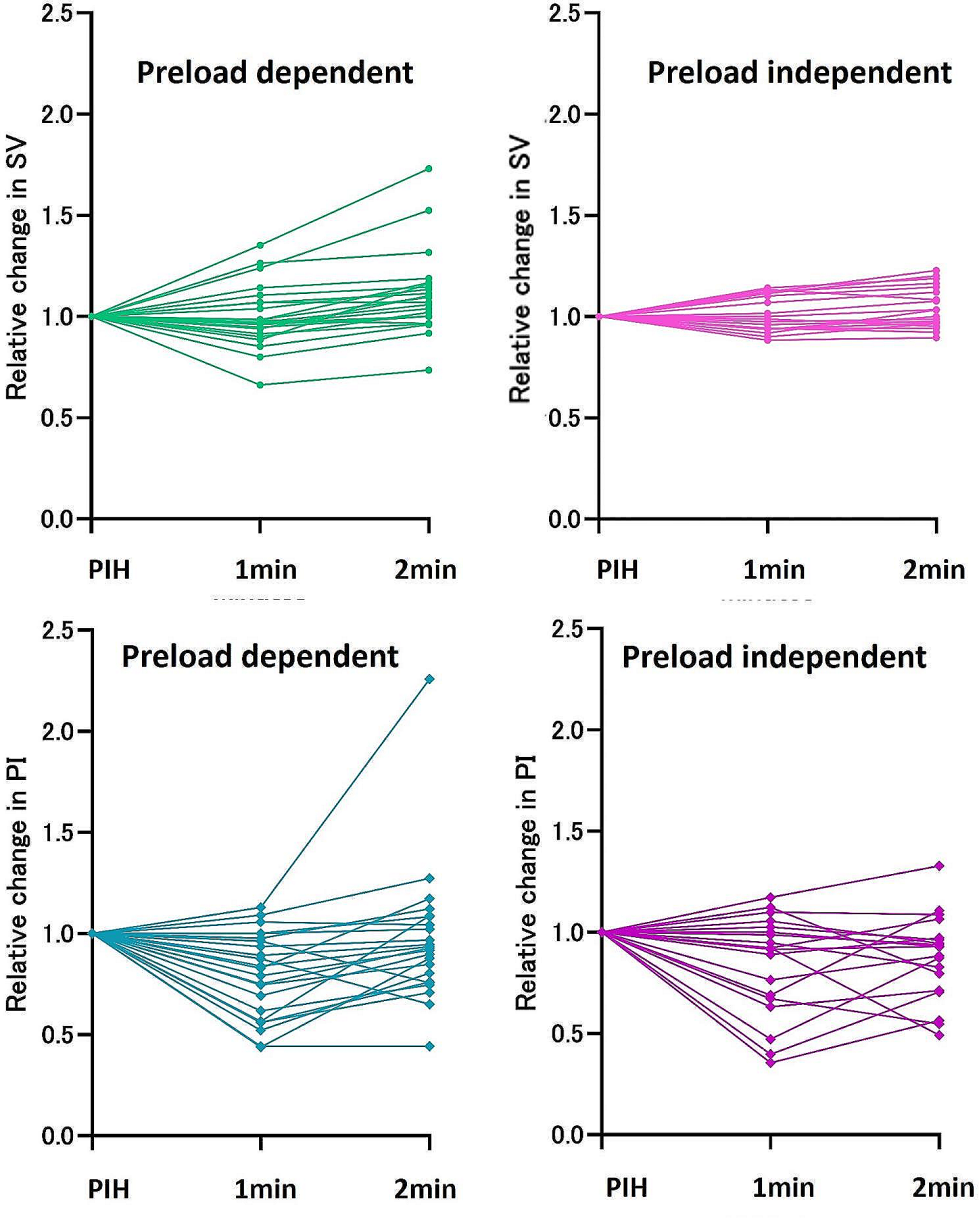
Relative changes in stroke volume and peripheral perfusion index in preload-dependent and preload-independent groups

A total of 50 patients who met the eligibility criteria were identified. Among these, 42 experienced hypotension during the observational period (between the induction of general anaesthesia and the skin incision) and were administered phenylephrine. The characteristics of these patients are presented in Table 1. For the 42 identified patients, haemodynamic data before and after phenylephrine administration were collected. SVV was higher in patients with preload dependency than in those without. Table 2 presents the changes in haemodynamic parameters induced by phenylephrine administration between post-induction hypotension and 1 and 2 min after phenylephrine administration within groups with and without preload dependency. The variables with significant differences within the groups are indicated. Table 3 presents the rate of change from post-induction hypotension to each time point for the main haemodynamic parameters within the groups. Figure 1 illustrates the changes in SV and PI from post-induction hypotension in all patients. Table 4 presents the predictability of preload dependency determined using the change in PI (the ratio of PI values between post-induction hypotension and 1 min after phenylephrine administration, the ratio of PI values between post-induction hypotension and 2 min after phenylephrine administration, and the ratio of PI values between 1 and 2 min after phenylephrine administration) and the predictability of other variables at post-induction hypotension. The relative changes in PI after the administration of phenylephrine at 1 min (ROC: 0.577, 95% confidence interval [CI]: 0.395–0.758) and 2 min (ROC: 0.557, 95% CI: 0.379–0.736) exhibited low accuracy for predicting preload dependency.

**Table 1:** Patient characteristics at post-induction hypotension

	Preload dependent	Preload independent	*P* value
Number	23	19	-
Age (years)	73 (58–75.5)	73 (66–76.5)	0.82
Sex male (n)	11	7	0.49
Height (cm)	160 ± 8.3	157 ± 9.6	0.22
Weight (kg)	58.4 ± 10.2	58.5 ± 9.8	0.96
Predicted body weight (kg)	56.7 ± 5.8	54.3 ± 6.5	0.22
Temperature of finger (℃)	36.4 (36.3–36.6)	36.3 (36.3–36.6)	0.72
Type of surgery (n)			0.28
Hepatobiliary and pancreatic surgery	2	6	
Gastrointestinal surgery	6	2	
Gynaecological surgery	6	6	
Urological surgery	4	1	
Vascular surgery	1	2	
Head and neck surgery	1	1	
Others	3	1	
Anaesthetic agents used for maintenance (n)			0.75
Propofol	16	12	
Remimazolam	1	1	
Sevoflurane	3	4	
Desflurane	3	2	
Bispectral Index	46 ± 10	47 ± 7.4	0.92
Tidal volume (mL)	462 ± 46	438 ± 55	0.13
Tidal volume (mL/predicted body weight)	8.1 (8.0–8.3)	8.2 (7.8–8.2)	0.34
Respiratory rate (cycles/min)	10 (10–12)	11 (10–12)	0.84
Minutes volume (L/min)	5.0 ± 0.8	4.7 ± 0.8	0.24
Heart rate (bpm)	68 ± 13	59 ± 9	0.01
Systolic arterial pressure (mmHg)	78 ± 9	89 ± 9	< 0.01
Diastolic arterial pressure (mmHg)	40 ± 5	40 ± 6	0.94
Mean arterial pressure (mmHg)	53 ± 6	57 ± 6	0.04
Pulse pressure (mmHg)	38 ± 8	49 ± 11	< 0.01
Cardiac output (L/min)	3.7 ± 0.9	3.9 ± 1.0	0.38
Cardiac index (L/min/BSA)	2.3 ± 0.6	2.5 ± 0.5	0.23
Stroke volume (mL)	55 ± 10	67 ± 19	< 0.01
Stroke volume index (mL/BSA)	34 ± 5.6	43 ± 8.6	< 0.01
Stroke volume variation (%)	17.8 ± 7.1	7.1 ± 2.4	< 0.01
Pulse pressure variation (%)	17.7 ± 8.0	7.0 ± 3.2	< 0.01
Perfusion index (%)	5.9 ± 2.9	7.8 ± 2.4	0.03
Pleth variability index (%)	15.5 ± 6.6	10.9 ± 7.0	0.04
Total peripheral resistance (dyne-sec/cm^5^)	1209 ± 272	1217 ± 301	0.93
Dynamic arterial elastance	0.99 ± 0.20	0.99 ± 0.29	0.99

**Table 2:** Effect of phenylephrine on hemodynamic values in the preload dependent and independent groups

		Preload dependent (*n* = 23)				Preload independent (*n* = 19)					
	Post-induction hypotension		1 min	2 min	*P* value		Post-induction hypotension	1 min	2 min	*P* value	
Heart rate (bpm)	68 ± 13		64 ± 11	60 ± 11	< 0.001		59 ± 9	55 ± 8	54 ± 8	< 0.001	
Systolic arterial pressure (mmHg)	78 ± 9		107 ± 21	123 ± 35	< 0.001		89 ± 9	121 ± 13	135 ± 20	< 0.001	
Diastolic arterial pressure (mmHg)	40 ± 5		57 ± 11	61 ± 15	< 0.001		40 ± 6	58 ± 13	61 ± 13	< 0.001	
Mean arterial pressure (mmHg)	53 ± 6		75 ± 15	83 ± 23	< 0.001		57 ± 6	79 ± 12	88 ± 16	< 0.001	
Pulse pressure (mmHg)	38 ± 8		51 ± 14	62 ± 22	< 0.001		49 ± 11	63 ± 13	74 ± 14	< 0.001	
Cardiac output (L/min)	3.7 ± 0.9		3.4 ± 0.5	3.5 ± 0.5	0.11		3.9 ± 1.0	3.7 ± 0.8	3.7 ± 0.9	0.04	
Cardiac index (L/min/BSA)	2.3 ± 0.6		2.1 ± 0.3	2.2 ± 0.4	0.04		2.5 ± 0.5	2.4 ± 0.4	2.4 ± 0.5	0.028	
Stroke volume (ml)	55 ± 10		54 ± 9	59 ± 9	0.01		67 ± 19	68 ± 18	70 ± 18	0.10	
Stroke volume index (mL/BSA)	34 ± 5.6		34 ± 4.9	37 ± 4.9	0.01		43 ± 8.6	43 ± 8.4	44 ± 8.5	0.09	
Stroke volume variation (%)	17.8 ± 7.1		17.7 ± 6.4	13.7 ± 6.7	< 0.001		7.1 ± 2.4	7.7 ± 2.5	6.8 ± 2.1	0.08	
Pulse pressure variation (%)	17.7 ± 8.0		17.7 ± 8.0	13.0 ± 7.5	< 0.001		7.0 ± 3.2	7.4 ± 2.9	6.8 ± 2.3	0.52	
Perfusion index (%)	5.9 ± 2.9		4.4 ± 2.2	5.1 ± 2.3	< 0.001		7.8 ± 2.4	6.4 ± 2.5	6.8 ± 2.5	0.007	
Pleth variability index (%)	15.5 ± 6.6		16.3 ± 8.3	17.5 ± 7.1	0.17		10.9 ± 7.0	9.4 ± 6.1	10.2 ± 5.7	0.20	
Total peripheral resistance (dyne-sec/cm^5^)	1209 ± 272		1798 ± 379	1953 ± 583	< 0.001		1217 ± 301	1811 ± 494	1989 ± 577	< 0.001	
Dynamic arterial elastance	0.99 ± 0.20		0.99 ± 0.21	0.95 ± 0.20	0.52		0.99 ± 0.29	0.92 ± 035	1.04 ± 025	0.24	

**Table 3:** Relative changes after phenylephrine administration

	Preload dependent (*n* = 23)	Preload independent (*n* = 19)	*P* value
**Relative change from post-induction hypotension to 1 min**			
Heart rate	0.946 ± 0.08	0.930 ± 0.05	0.48
Systolic arterial pressure	1.383 ± 0.22	1.371 ± 0.18	0.85
Diastolic arterial pressure	1.412 ± 0.24	1.448 ± 0.26	0.64
Mean arterial pressure	1.409 ± 0.24	1.386 ± 0.19	0.74
Pulse pressure	1.356 ± 0.21	1.304 ± 0.17	0.40
Cardiac output	0.947 ± 0.17	0.941 ± 0.10	0.89
Stroke volume	0.999 ± 0.15	1.010 ± 0.09	0.77
Stroke volume variation	1.001 ± 0.09	1.139 ± 0.36	0.10
Pulse pressure variation	1.017 ± 0.15	1.113 ± 0.25	0.13
Perfusion Index	0.794 ± 0.21	0.844 ± 0.25	0.49
Pleth variability index	1.080 ± 0.40	0.981 ± 0.56	0.51
Total peripheral resistance	1.56 ± 0.53	1.50 ± 0.31	0.64
Dynamic arterial elastance	1.01 ± 0.13	1.01 ± 0.23	0.99
**Relative change from post-induction hypotension to 2 min**			
Heart rate	0.888 ± 0.10	0.916 ± 0.56	0.28
Systolic arterial pressure	1.583 ± 0.43	1.530 ± 0.27	0.64
Diastolic arterial pressure	1.532 ± 0.39	1.527 ± 0.27	0.96
Mean arterial pressure	1.570 ± 0.42	1.545 ± 0.27	0.79
Pulse pressure	1.649 ± 0.54	1.540 ± 0.28	0.43
Cardiac output	0.985 ± 0.22	0.959 ± 0.11	0.64
Stroke volume	1.104 ± 0.20	1.047 ± 0.10	0.27
Stroke volume variation	0.768 ± 0.22	0.985 ± 0.20	0.002
Pulse pressure variation	0.768 ± 0.31	1.087 ± 0.38	0.005
Perfusion Index	0.967 ± 0.33	0.877 ± 0.21	0.32
Pleth variability index	1.188 ± 0.35	1.180 ± 0.90	0.97
Total peripheral resistance	1.74 ± 0.82	1.64 ± 0.35	0.62
Dynamic arterial elastance	0.98 ± 0.22	1.11 ± 0.31	0.15

**Table 4:** Ability to predict preload dependency

Index	Area under the ROC curve	95% confidence interval	Cut-off value	Sensitivity	Specificity	Youden index
PI at PIH	0.706	0.545–0.867	6.2	0.789	0.652	0.44
ΔPI 0–1 min	0.577	0.395–0.758	0.875	0.609	0.632	0.24
ΔPI 0–2 min	0.557	0.379–0.736	1.021	0.348	0.789	0.14
ΔPI 1–2 min	0.664	0.489–0.838	1.021	0.826	0.526	0.35
SV at PIH	0.773	0.626–0.921	57	0.652	0.842	0.49
ΔSV 0–1 min	0.54	0.36–0.72	0.984	0.652	0.526	0.18
ΔSV 0–2 min	0.579	0.401–0.757	1.00	0.783	0.421	0.20
ΔSV 1–2 min	0.77	0.628–0.912	1.078	0.565	0.895	0.46
SAP at PIH	0.831	0.702–0.959	78	0.609	0.947	0.56
MAP at PIH	0.698	0.528–0.868	53	0.609	0.789	0.40
PP at PIH	0.795	0.658–0.932	43	0.826	0.684	0.51
CO at PIH	0.481	0.296–0.666	3.64	0.652	0.526	0.18
CI at PIH	0.593	0.418–0.767	1.8	0.261	1.000	0.26
SVI at PIH	0.834	0.706–0.963	36	0.783	0.842	0.63
PVI at PIH	0.707	0.542–0.872	9	0.913	0.474	0.39
PPV at PIH	0.954	0.894–1.000	11	0.870	0.947	0.82

PIH: post-induction hypotension; Δ 0–1 min: relative change from post-induction hypotension to 1 min; Δ 0–2 min: relative change from post-induction hypotension to 2 min; Δ 1-2 min: relative change from 1 to 2 min; ROC: receiver operating curve; PI: peripheral perfusion index; SV: stroke volume; SBP: systolic arterial pressure; MAP: mean arterial pressure; PP: pulse pressure; CO: cardiac output; CI: cardiac index; SV: stroke volume; SVI: stroke volume index; PVI: pleth variability index; PPV: pulse pressure variation

**Fig. 2:**
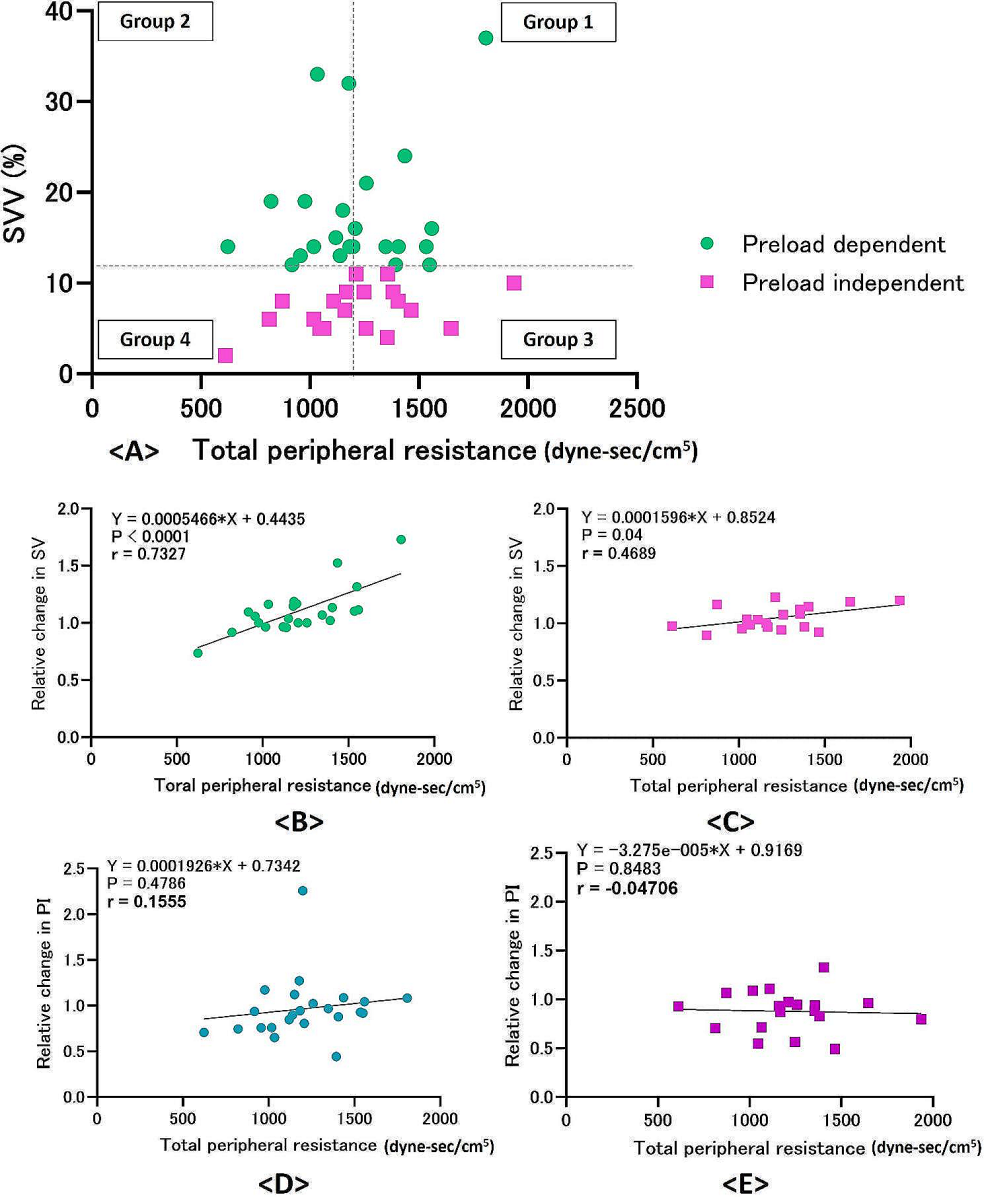
Relationship between total peripheral resistance at post-induction hypotension and relative change in stroke volume after phenylephrine administration. (**A**) Scatter diagram of total peripheral resistance at post-induction hypotension and stroke volume variation. (**B**) Correlation between total peripheral resistance at post-induction hypotension and relative change in stroke volume between post-induction hypotension and 2 min after phenylephrine administration in patients with preload dependency. (**C**) Correlation between total peripheral resistance at post-induction hypotension and relative change in stroke volume between post-induction hypotension and 2 min after phenylephrine administration in patients with preload independency. (**D**) Correlation between total peripheral resistance at post-induction hypotension and relative change in perfusion index between post-induction hypotension and 2 min after phenylephrine administration in patients with preload dependency. (**E**) Correlation between total peripheral resistance at post-induction hypotension and relative change in stroke volume between post-induction hypotension and 2 min after phenylephrine administration in patients with preload independency

Regarding afterload, we further analysed the relationship between increased SV and post-induction hypotension 2 min after phenylephrine administration and TPR at post-induction hypotension, revealing that TPR at post-induction hypotension was widely distributed regardless of preload dependency (Fig. 2A), with high correlation between TPR at post-induction hypotension and SV increase only observed in patients with preload dependency (*r* = 0.73) (Fig. 2B and C). There was no correlation between TPR at post-induction hypotension and relative change in PI after phenylephrine administration in groups with and without preload dependency (Fig. 2D and E). Subgroup analysis was based on preload dependency and the TPR values according to a threshold of 1179 dyne-sec/cm^5^ (Fig. 2A).

**Fig. 3:**
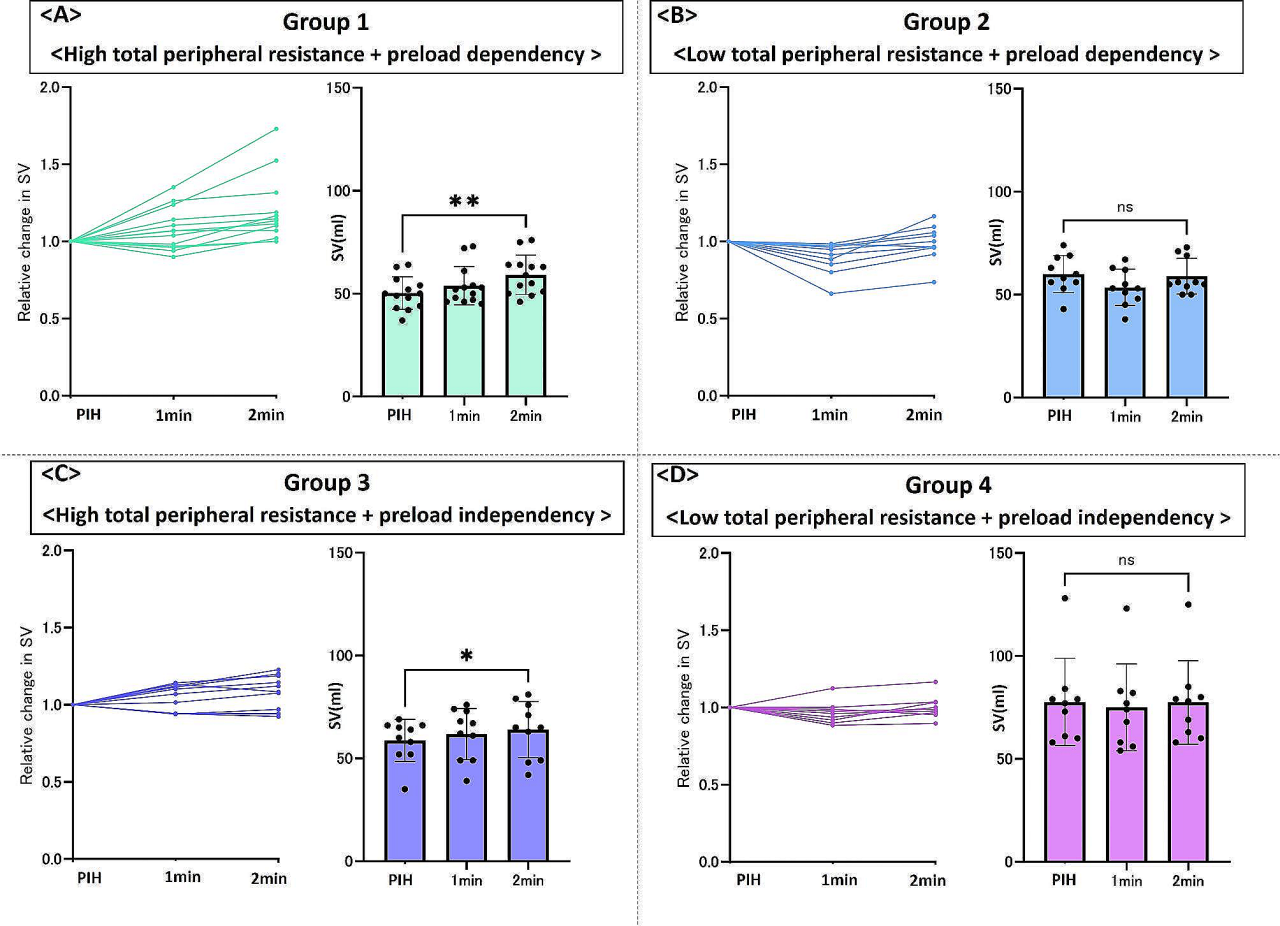
Relative and absolute change in the stroke volume after phenylephrine administration. (**A**) Relative and absolute change in the stroke volume in patients in Group (1) (**B**) Relative and absolute change in the stroke volume in patients in Group (2) (**C**) Relative and absolute change in the stroke volume in patients in Group (3) (**D**) Relative and absolute change in the stroke volume in patients in Group (4) PIH, post-induction hypotension. **P < 0.01, *P < 0.05

**Fig. 4:**
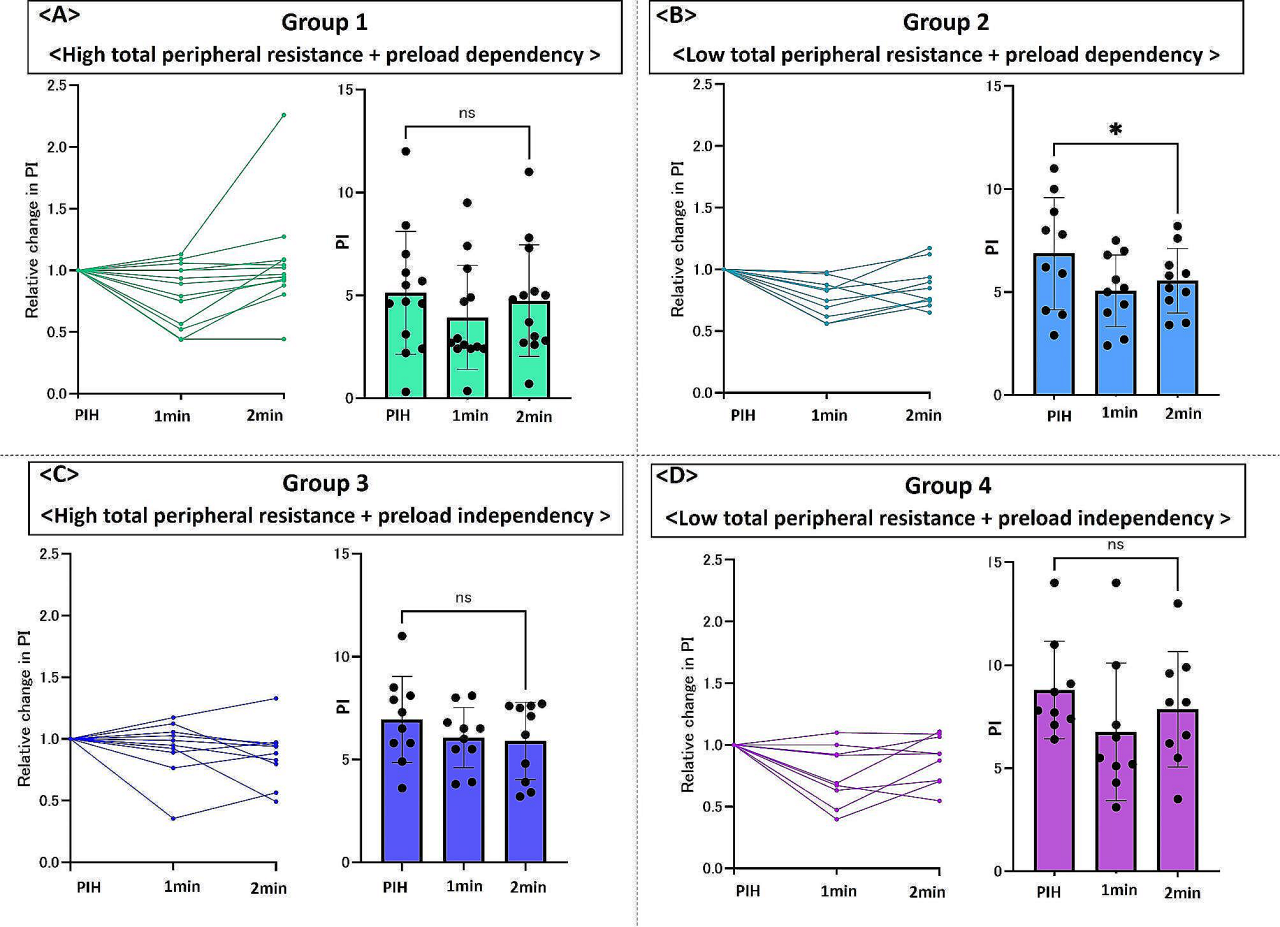
Relative and absolute change in the perfusion index after phenylephrine administration. (**A**) Relative and absolute change in perfusion index in patients in Group (1) (**B**) Relative and absolute change in perfusion index in patients in Group (2) (**C**) Relative and absolute change in perfusion index in patients in Group (3) (**D**) Relative and absolute change in perfusion index in patients in Group (4) PIH, post-induction hypotension. *P < 0.05

Figure 3 shows the relative and absolute changes in SV in patients in each group divided according to preload dependency and the TPR value. In Group 1, SV was significantly increased (*n* = 13) (50.2 ± 7.9 mL at post-induction hypotension to 59.2 ± 9.5 mL 2 min after phenylephrine administration, *P* = 0.003) (Fig. 3A). In Group 2, SV tended to decrease, but no significant difference was observed (*n* = 10) (60.0 ± 9.0 mL vs. 58.9 ± 8.7 mL, respectively; *P* = 0.64) (Fig. 3B). In Group 3, SV was significantly increased but also tended to decrease (*n* = 10) (58.8 ± 10.2 mL vs. 63.9 ± 13.6 mL, respectively; *P* = 0.03) (Fig. 3C). In Group 4, SV remained unchanged or appeared to decrease slightly (*n* = 9) (77.7 ± 21.1 mL vs. 77.4 ± 20.3 mL, respectively; *P* = 0.9) (Fig. 3D). The patterns of SV change induced by phenylephrine varied across the groups. Figure 4 presents the relative and absolute changes in PI in patients in each group divided according to preload dependency and the TPR value. In Group 2, PI was significantly decreased (6.9 ± 2.7% at post-induction hypotension to 5.6 ± 1.6% 2 min after phenylephrine administration, *P* = 0.012) (Fig. 4B), while PI did not differ significantly in other groups. Supplemental Table 1 shows the changes in hemodynamic parameters induced by phenylephrine in each group.

## Discussion

In the present study, the change in PI induced by phenylephrine administration could not predict preload dependency during general anaesthesia. Unsurprisingly, PPV demonstrated high predictability for preload dependency at post-induction hypotension, given that SVV and PPV are closely coupled. SAP, PP, and SVI could also moderately predict preload dependency.

To the best of our knowledge, this is the first study to investigate the ability of the changes in PI induced by phenylephrine administration to predict preload dependency. Although our results failed to validate the hypothesis that changes in PI after phenylephrine administration could predict the preload dependency, we revealed new insights indicating that phenylephrine-induced changes in SV exhibit various patterns according to preload dependency and afterload.

These negative results could be attributed to several possible reasons. First, the post-induction hypotension status varied, indicating the occurrence of more complex phenylephrine effects than anticipated. The primary cause of post-induction hypotension is arterial dilation with reduced systemic vascular resistance and venous dilation with decreased venous return [17]. If the degree of vasodilation is severe, the patients experience preload dependency due to the reduced SV. Following phenylephrine administration, the increase in venous return is higher when the degree of vasodilation is higher. In addition to changes in venous return, the afterload status mediates the phenylephrine effect. TPR was selected as a parameter of afterload instead of systemic vascular resistance (SVR) because we did not measure central venous pressure (CVP). However, the impact of differences between SVR and TPR on our results appears limited, given that CVP may exhibit similar values at post-induction hypotension. Our results showed that TPR at post-induction hypotension was widely distributed in patients with and without preload dependency and strongly correlated with increased SV after phenylephrine administration in groups with preload dependency (Fig. 2A and B). This indicates that phenylephrine increased SV with high TPR and decreased SV with low TPR in patients with preload dependency. TPR values may explain the wide variations in SV changes after phenylephrine administration, especially in patients with preload dependency.

Preload dependency and TPR seemed important factors to clarify the effect of phenylephrine on SV. Accordingly, we generated four subgroups according to the TPR value and preload dependency to explain our findings. In general, if patients present the same MAP value, high TPR indicates small CO, while low TPR indicates high CO. Preload dependency also implies a relatively small SV. Therefore, in our subgroups, SV was speculated to be smallest in Group 1 (high TPR with preload dependency) and largest in Group 4 (low TPR and preload independency), although our patients exhibited varied MAP values at post-induction hypotension (53 ± 6 mmHg in preload-dependent patients, 57 ± 6 mmHg in preload-independent patients, respectively).

**Fig. 5:**
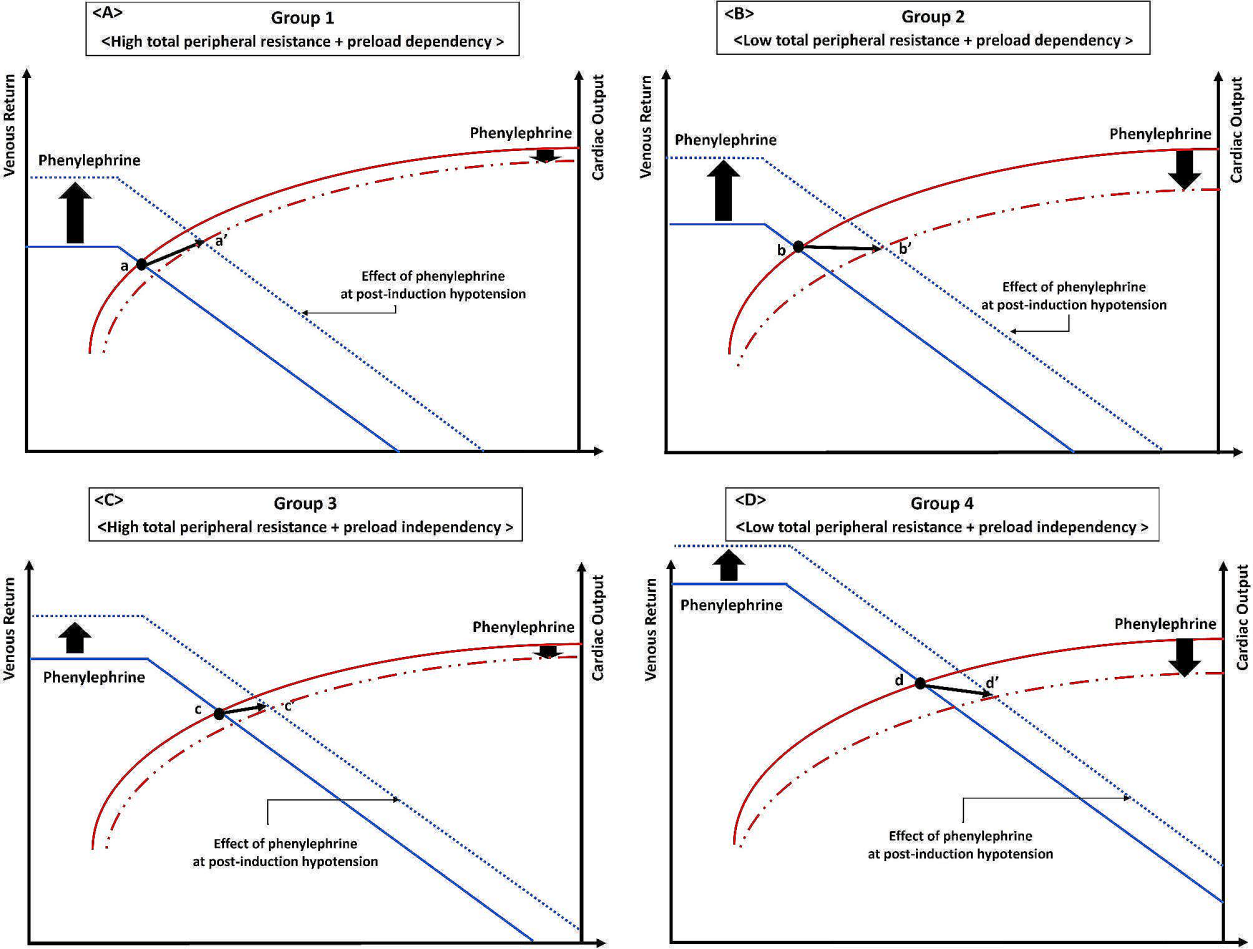
Theoretical model explaining different patterns of change in stroke volume after phenylephrine administration. (**A**) Venous return (left) and cardiac output (right) during post-induction hypotension and after phenylephrine administration in patients with high total peripheral resistance and preload dependency (Group 1). The crossing point of the respective venous return and SV curves indicates the instantaneous working point of the heart during post-induction hypotension (a). The new crossing point a’ is observed after the administration of phenylephrine. (**B**) The crossing point of the respective venous return and SV curves indicates the instantaneous working point of the heart at post-induction hypotension (b) in patients with low total peripheral resistance and preload dependency (Group 2). The new crossing point b’ is observed after the administration of phenylephrine. (**C**) The crossing point of the respective venous return and SV curves indicates the instantaneous working point of the heart at post-induction hypotension (c) in patients with high total peripheral resistance and preload independency (Group 3). The new crossing point c’ is observed after the administration of phenylephrine. (**D**) The crossing point of the respective venous return and SV curves indicates the instantaneous working point of the heart at post-induction hypotension (d) in patients with low total peripheral resistance and preload independency (Group 4). The new crossing point d’ is observed after the administration of phenylephrine. SV, stroke volume

Figure 5 represents a theoretical model to explain different patterns of change in SV after phenylephrine administration. In this theoretical model, the degree of venous return induced by phenylephrine was assumed to be larger in the preload-dependent group than in the preload-independent group, as the degree of vasodilation may be greater in the preload-dependent group supported by lower MAP and smaller SV; however, the mean TPR value was similar between groups with and without preload dependency. In Group 1, patients exhibited the smallest SV. Phenylephrine induced a larger volume of venous return in patients with preload dependency than in those without preload dependency and the increase in afterload may have been relatively small, given the previously elevated TPR. In fact, the relative change in TPR between post-induction hypotension and 2 min after phenylephrine administration was smaller in patients with high TPR (Group 1 + 3) than in those with low TPR (Group 2 + 4) (1.35 ± 0.25 vs. 1.76 ± 0.52, respectively; *P* = 0.002). This pattern resulted in an increase in SV (a-a’ in Fig. 5A). In Group 2, patients also exhibited small SV, but it was relatively higher than that of patients in Group 1. The increase in venous return may be similar to that observed in Group 1, while the increase in afterload was relatively high, resulting in an unchanged or potentially decreased SV pattern (b-b’ in Fig. 5B). In Group 3, patients had high TPR but higher SV than patients in Group 1. The effect of phenylephrine on venous return was smaller in Group 3 than in Group 1 owing to preload independency, while afterload was similar to that of Group (1) The small increase in SV could be attributed to the small increase in venous return and to the fact that SV had already reached a place where the slope of the venous return and cardiac function curves was slower or plateaued (c-c’ in Fig. 5C). In Group 4, patients exhibited the largest SV. The effect of phenylephrine on venous return was smaller in Group 4 than in Group 2, while the increase in afterload was relatively high, as shown in Group (2) SV remained unchanged or slightly decreased after phenylephrine administration since the effect of increased afterload may overcome the slightly increased SV due to the slower slope of the venous return and cardiac function curves (d-d’ in Fig. 5D). These simple models can explain the different patterns of change in SV in patients with preload dependency. Less variation in SV change in patients without preload decency may be attributed to differences in the slope of venous return and cardiac function curves. In this model, relative and absolute values of PI did not follow the patterns in SV (Fig. 4).

Our results differed from a study by Rebet et al., where SV in patients with preload dependency was unchanged but significantly decreased in patients without preload dependency after phenylephrine administration [9]. The authors revealed that SV at post-induction hypotension was relatively smaller in patients with preload dependency, and afterload tended to be higher in patients with preload dependency when compared with our results (with a systemic vascular resistance index [SVRI; dyne-sec/cm^5^m^2^] of 2019 [IQR 1255; 2540], 1809 [IQR 1394; 2445] in the preload-dependent and -independent groups, respectively). In our results, SVRI (calculated as they did [MAP/CI × 80]) was 1867 (IQR 1635; 2291) and 1800 (1683; 2145) in the preload-dependent and -independent groups, respectively. This implies that more patients in the study by Rebet et al. were classified into Group 1 than in our study. In Group 1, SV should theoretically increase rather than decrease; however, SV in patients with preload dependency did not change, whereas SV in patients with preload independency significantly decreased. The observed difference could be attributed to the phenylephrine dose employed. Rebet et al. used 0.05 − 0.15 mg phenylephrine based on the extent of hypotension. More patients with preload dependency were speculated to have received a larger dose of phenylephrine than patients with preload independency. A larger phenylephrine dose can hinder the increase in venous return owing to a more pronounced increase in afterload, even when classified in Group 1. In patients with preload independency, a smaller phenylephrine dose may lead to a smaller increase in venous return, resulting in decreased SV due to increased afterload. Given that the authors did not identify changes in SV for individual cases, further speculation is not possible. However, a more detailed comparison with our study could have been possible had the authors created four divided models accounting for changes in both preload and afterload, as performed in the current study.

The relative changes in SV and PI, as surrogate parameters of SV, could not predict preload dependency accurately (Table 4). The degree of increase in SV after phenylephrine administration varied in the preload-dependent group owing to distinct patterns of phenylephrine effects according to the status of preload dependency and TPR in the current study. We believe that this is the main reason underlying the failure to predict preload dependency using the change in PI.

Second, PI might be more affected by peripheral vascular tone than the change in SV. During anaesthesia, PI increases in vasoplegia owing to relatively unchanged SV with decreased vascular tone, whereas PI decreases in hypovolemia and cardiac failure because of decreased SV with increased vascular tone [4]. The absolute value of PI is considered to not reflect SV owing to wide inter-patient variations [18, 19]. In fact, we found no strong correlation between the absolute values of PI and SV at post-induction hypotension (*r* = 0.481). Therefore, we focused on the relative ratio of PI, which may serve as a surrogate parameter for changes in SV. Højlund et al. demonstrated a good correlation between the relative change in SV and PI and the supine position and the head-up tilt position, as well as between before and after phenylephrine administration in the head-up tilt position (*r* = 0.7 and 0.6, respectively) [5]. The authors administered 0.1 or 0.2 mg of phenylephrine until the SVV was < 12% in the head-up tilt position to mimic hypovolemia and evaluated PI upon reaching the hemodynamically stable state. However, contrary to expectations, the correlation between relative change in PI from post-induction hypotension and change in SV was also poor at 1 and 2 min (*r* = 0.394 and 0.331, respectively). These differences could be explained by the influence of vascular tone on PI. Højlund et al. evaluated hemodynamic parameters, including PI at a hemodynamically stable state, such as 3 min after position change; however, our intervention and measurements were conducted during the post-induction period when MAP decreased to < 65 mmHg. In general, haemodynamic parameters are unstable during this period. Most of our patients received phenylephrine during an ongoing MAP drop; however, we could not delay intervention until the MAP had fallen and stabilized to a very low level. In situations where the peripheral vascular tone is unstable, PI may not reflect the change in SV. The timing of measurement of the first set (at post-induction hyperextension) could have impacted our results.

In addition, PI may be evaluated after an adequate period of phenylephrine administration. In an animal study, the increase in afterload was found to occur first, followed by the increase in venous return after administering phenylephrine [20]. SVV and PPV at 1 min after phenylephrine administration did not change from post-induction hypotension in the preload-dependent group (Table 2), supporting the order of response to phenylephrine. Therefore, the change in PI may be primarily attributed to a change in vascular tone and not SV 1 min after phenylephrine administration. In most patients, changes in SV at 2 min appeared stable after peak effects of phenylephrine. However, PI may remain in an unstable state owing to increased peripheral vascular tone, preventing the use of PI as an alternative to SV. In fact, the relative change in PI differs significantly from the relative change from that post-induction hypotension to each time point in SV after phenylephrine administration (1 min after administration; 0.82 ± 0.22 vs. 1.00 ± 0.13, *P* < 0.0001, 2 min after administration; 0.93 ± 0.29 vs. 1.08 ± 0.17, respectively; *P* = 0.004,). Phenylephrine reduced PI when compared with SV, even 2 min after phenylephrine administration. As shown in Fig. 4, the mean PI value was not increased in any subgroups after phenylephrine administration. These results imply that PI could be more affected by phenylephrine-induced peripheral vascular tone than initially hypothesized, where we speculated that PI would increase owing to increased venous return overcoming increased vascular tone. Moreover, the greater influence of phenylephrine-induced peripheral vascular tone on PI could explain why PVI did not decrease in the preload-dependent group 2 min after phenylephrine administration, although SVV PPV were decreased significantly.

Based on these results, the interpretation of PI as an alternative to SV should be interpreted with caution in situations where vascular resistance increases rapidly. The value of PI may be evaluated at a hemodynamically stable state, warranting more than 2 min to evaluate the change in SV in those situations.

This study introduced new models of SV changes after phenylephrine administration at post-induction hypotension. In preload-dependent patients, SV both increased and decreased according to TPR. An increase in SV could be obtained only in patients with high TPR, although phenylephrine increased MAP to maintain tissue perfusion. Hence, phenylephrine should be used with caution in patients with low TPR as it may lead to decreased SV.

We opted to use phenylephrine as a vasopressor instead of norepinephrine based on local regulations, given the high rate of phlebitis reported following norepinephrine administered via a peripheral intravascular catheter [21]. Further research to determine the effect of norepinephrine on changes in SV at post-induction hypotension is warranted.

Eadyn, another parameter of arterial tone, reportedly correlates with MAP responsiveness (MAP increase after fluid bolus) when high but not when low [22]. Eadyn did not significantly differ between groups with and without preload dependency during post-induction hypotension (0.92 ± 0.17 and 1.07 ± 0.21 in Groups 1 and 2, respectively). A low Eadyn in Group 1 may imply that both fluid bolus and vasopressor are needed to treat hypotension; however, Eadyn was relatively higher in Group 2 than in Group 1, indicating that fluid bolus alone could optimize the SV and MAP. Further research is needed to interpret Eadyn at post-induction hypotension.

This study has several limitations. First, preload dependency (fluid responsiveness) was determined using SVV values rather than actual fluid administration. The tidal volume was set as 8 mL/PBW, and patients with conditions that affected the interpretation of SVV were excluded to increase accuracy. Although we set a threshold of 12% for preload dependency, a recent meta-analysis on the ability of SVV to predict preload dependency has suggested an optimal threshold of 10.7% in patients with closed chest and abdomen [23]. Thus, if we had used an alternate threshold, our result may have differed. Second, the choice of anaesthetic agents used to maintain general anaesthesia was not controlled. The type of anaesthetic used in the study cases could have impacted PI, as the degree of vasodilation differed between anaesthetics. However, patients with intraoperative hypotension had varying degrees of vasodilation-mixed hypovolaemia and the influence of the type of anaesthetic agent on the results may have been minimal in this pragmatic study. Third, the value of PI in various clinical settings may differ between manufacturers owing to different algorithms to obtain photoplethysmography. The observed findings may vary if a system other than Masimo technology (Masimo Corp, Irvine, CA, USA) is employed, although PI performance between manufacturers has never been evaluated. Fourth, the phenylephrine-induced increase in venous return may differ in patients with hypovolemia. If patients appear to be hypovolemic before anaesthetic induction, the increase in venous return after phenylephrine administration during post-induction hypotension tends to be much smaller than in patients without hypovolemia, resulting in decreased SV. Haemodynamic data pre-anaesthetic induction could have helped distinguish hypovolemic patients before anaesthesia induction and enabled a more detailed analysis of the effect of phenylephrine. However, such data was unavailable, given that we inserted the arterial line after anaesthetic induction in most patients. Moreover, we only evaluated data during the post-induction period. If patients become hypovolemic owing to intraoperative haemorrhage, the administration of phenylephrine may decrease SV by increasing the venous return minimally and enhancing afterload markedly. In such scenarios, phenylephrine may be unsuitable. Lastly, our results may be inapplicable where a dose of phenylephrine other than 0.1 mg is used, given that larger or smaller doses of phenylephrine may show a different pattern of change in SV depending on the hemodynamic status of patients on the venous return and cardiac functional curve.

## Conclusions

The change in PI induced by administering 0.1 mg of phenylephrine was unable to predict preload dependency. This may be attributed to the variability in the effect of phenylephrine on SV in patients with preload dependency owing to the complex combination of vasodilation, TPR, and CO changes. SV change induced by phenylephrine could be explained by the four models divided according to preload dependency and TPR. The pattern of SV change differed with the degree of TPR and was more pronounced in patients with preload dependency.

### Electronic supplementary material

Below is the link to the electronic supplementary material.

Supplementary Material 1

## Data Availability

The data presented in this study are available on request from the corresponding author.

## Abbreviations

CI: Cardiac index
CO: Cardiac output
DAP: Diastolic arterial pressure
Eadyn: Dynamic arterial elastance
HR: Heart rate
MAP: Mean arterial pressure
PBW: Predicted body weight
PI: Peripheral perfusion index
PP: Pulse pressure
PPV: Pulse pressure variation
PVI: Pleth variability index
ROC: Receiver operating characteristic
SAP: Systolic arterial pressure
SV: Stroke volume
SVI: Stroke volume index
SVR: Systemic vascular resistance
SVRI: Systemic vascular resistance index
SVV: Stroke volume variation
TPR: Total peripheral resistance
