# Supplementary material for: Prediction of preload dependency using phenylephrine-induced peripheral perfusion index during general anaesthesia: a prospective observational study
Source: BMC Anesthesiol. 2024 Mar 2;24:88. doi: 10.1186/s12871-024-02478-w (PMC10908037; doi:10.1186/s12871-024-02478-w)
Supplement: Supplementary file 1 — Supplementary Material 1 [file 12871_2024_2478_MOESM1_ESM.docx]

Supplemental Table 1: Effect of phenylephrine on hemodynamic values in each subgroup

| **Group 1 (n=13)** | **Post-induction  hypotension** | **1 min** | **2 min** | **P value** |
| --- | --- | --- | --- | --- |
| **Heart rate (bpm)** | 64 ± 12 | 62 ± 13 | 59 ± 13 | 0.07 |
| **Systolic arterial pressure (mmHg)** | 81 ± 9 | 105 ± 18 | 115 ± 29 | <0.001 |
| **Diastolic arterial pressure (mmHg)** | 41 ± 5 | 54 ± 10 | 56 ± 13 | <0.001 |
| **Mean arterial pressure (mmHg)** | 54 ± 6.2 | 72 ± 13 | 76 ± 19 | <0.001 |
| **Cardiac output (L/min)** | 3.2 ± 0.7 | 3.3 ± 0.6 | 3.4 ± 0.6 | 0.16 |
| **Cardiac index (L/min/BSA)** | 2.0 ± 0.4 | 2.0 ± 0.4 | 2.2 ± 0.4 | 0.19 |
| **Stroke volume (ml)** | 50 ± 8 | 54 ± 9.4 | 59 ± 9.5 | 0.003 |
| **Stroke volume index (mL/BSA)** | 31 ± 5.8 | 34 ± 35.7 | 37 ± 5.8 | 0.003 |
| **Stroke volume variation (%)** | 18.5 ± 8.0 | 18.3 ± 6.8 | 14.4 ± 7.0 | 0.002 |
| **Pulse pressure variation (%)** | 16.9 ± 7.5 | 17.0 ± 7.1 | 13.0 ± 7.1 | 0.006 |
| **Perfusion index (%)** | 5.1 ± 3.0 | 3.9 ± 2.5 | 4.7 ± 2.7 | 0.21 |
| **Pleth variability index (%)** | 16.9 ± 7.3 | 16.3 ± 7.1 | 17.6 ± 6.7 | 0.43 |
| **Total peripheral resistance** | 1389 ± 189 | 1797 ± 408 | 1834 ± 533 | 0.015 |
| **Dynamic arterial elastance (dyne-sec/cm^5^)** | 0.92 ± 0.17 | 0.93 ± 0.14 | 0.92 ± 0.22 | 0.97 |
|  |  |  |  |  |
| **Group 2 (n=10)** | **Post-induction  hypotension** | **1 min** | **2 min** | **P value** |
| **Heart rate (bpm)** | 73 ± 12 | 67 ± 9 | 61 ± 8 | <0.001 |
| **Systolic arterial pressure (mmHg)** | 74 ± 7 | 111 ± 25 | 134 ± 40 | <0.001 |
| **Diastolic arterial pressure (mmHg)** | 39 ± 4 | 60 ± 11 | 68 ± 17 | <0.001 |
| **Mean arterial pressure (mmHg)** | 52 ± 4.3 | 79 ± 16 | 93 ± 26 | <0.001 |
| **Cardiac output (L/min)** | 4.3 ± 0.7 | 3.5 ± 0.3 | 3.6 ± 0.4 | 0.017 |
| **Cardiac index (L/min/BSA)** | 2.7 ± 0.4 | 2.1 ± 0.3 | 2.3 ± 0.4 | 0.025 |
| **Stroke volume (mL)** | 60 ± 9 | 54 ± 8.8 | 59 ± 8.7 | 0.64 |
| **Stroke volume index (mL/BSA)** | 37 ± 3.1 | 33 ± 3.8 | 37 ± 3.8 | 0.73 |
| **Stroke volume variation (%)** | 17.0 ± 6.2 | 17.0 ± 6.0 | 12.8 ± 6.5 | 0.006 |
| **Pulse pressure variation (%)** | 18.7 ± 8.9 | 18.6 ± 9.1 | 13.0 ± 8.4 | 0.025 |
| **Perfusion index (%)** | 6.9 ± 2.7 | 5.1 ± 1.7 | 5.6 ± 1.6 | 0.012 |
| **Pleth variability index (%)** | 13.6 ± 5.2 | 16.3 ± 10.1 | 17.3 ± 8.0 | 0.128 |
| **Total peripheral resistance** | 975 ± 161 | 1800 ± 358 | 2108 ± 635 | <0.001 |
| **Dynamic arterial elastance (dyne-sec/cm^5^)** | 1.07 ± 0.21 | 1.06 ± 0.17 | 0.99 ± 0.16 | 0.3 |
|  |  |  |  |  |
| **Group 3 (n=10)** | **Post-induction  hypotension** | **1 min** | **2 min** | **P value** |
| **Heart rate (bpm)** | 58 ± 9 | 54 ± 10 | 53 ± 9 | <0.001 |
| **Systolic arterial pressure (mmHg)** | 89 ± 8 | 122 ± 13 | 137 ± 18 | <0.001 |
| **Diastolic arterial pressure (mmHg)** | 43 ± 5 | 62 ± 12 | 64 ± 9 | <0.001 |
| **Mean arterial pressure (mmHg)** | 59 ± 3.4 | 81 ± 8.5 | 91 ± 11 | <0.001 |
| **Cardiac output (L/min)** | 3.4 ± 0.4 | 3.3 ± 0.39 | 3.3 ± 0.4 | 0.63 |
| **Cardiac index (L/min/BSA)** | 2.2 ± 0.3 | 2.2 ± 0.3 | 2.2 ± 0.3 | 0.42 |
| **Stroke volume (mL)** | 59 ± 10 | 62 ± 12 | 64 ± 14 | 0.031 |
| **Stroke volume index (ml/BSA)** | 38 ± 5.4 | 40 ± 6.6 | 42 ± 7.6 | 0.03 |
| **Stroke volume variation (%)** | 7.9 ± 2.6 | 8.1 ± 2.8 | 7.4 ± 2.1 | 0.24 |
| **Pulse pressure variation (%)** | 7.6 ± 3.7 | 8.1 ± 3.1 | 7.3 ± 2.5 | 0.7 |
| **Perfusion index (%)** | 6.9 ± 2.1 | 6.1 ± 1.5 | 5.9 ± 1.9 | 0.11 |
| **Pleth variability index (%)** | 11.7 ± 6.1 | 8.9 ± 5.0 | 9.3 ± 4.7 | 0.17 |
| **Total peripheral resistance** | 1426 ± 218 | 2003 ± 354 | 2241 ± 401 | <0.001 |
| **Dynamic arterial elastance (dyne-sec/cm^5^)** | 0.95 ± 0.3 | 1.01 ± 0.28 | 1.01 ± 0.26 | 0.6 |
|  |  |  |  |  |
| **Group 4 (n=9)** | **Post-induction  hypotension** | **1 min** | **2 min** | **P value** |
| **Heart rate (bpm)** | 60 ± 9 | 55 ± 6 | 55 ± 6 | 0.01 |
| **Systolic arterial pressure (mmHg)** | 90 ± 11 | 120 ± 14 | 133 ± 23 | <0.001 |
| **Diastolic arterial pressure (mmHg)** | 38 ± 7 | 54 ± 14 | 58 ± 16 | 0.002 |
| **Mean arterial pressure (mmHg)** | 55 ± 7.6 | 77 ± 15 | 84 ± 21 | <0.001 |
| **Cardiac output (L/min)** | 4.6 ± 1.0 | 4.1 ± 1.0 | 4.2 ± 0.9 | 0.117 |
| **Cardiac index (L/min/BSA)** | 2.8 ± 0.5 | 2.6 ± 0.5 | 2.6 ± 0.5 | 0.07 |
| **Stroke volume (mL)** | 78 ± 21 | 75 ± 21 | 77 ± 20 | 0.9 |
| **Stroke volume index (ml/BSA)** | 47 ± 9.4 | 46 ± 9.7 | 47 ± 9.2 | 0.85 |
| **Stroke volume variation (%)** | 6.2 ± 2.1 | 7.2 ± 2.2 | 6.1 ± 2.1 | 0.82 |
| **Pulse pressure variation (%)** | 6.3 ± 2.7 | 6.6 ± 2.3 | 6.3 ± 2.1 | 1 |
| **Perfusion index (%)** | 8.8 ± 2.4 | 6.8 ± 3.3 | 7.9 ± 2.8 | 0.09 |
| **Pleth variability index (%)** | 10.1 ± 8.2 | 10.0 ± 7.3 | 11.2 ± 6.8 | 0.38 |
| **Total peripheral resistance** | 985 ± 184 | 1599 ± 558 | 1709 ± 632 | 0.002 |
| **Dynamic arterial elastance (dyne-sec/cm^5^)** | 1.02 ± 0.3 | 0.91 ± 0.22 | 1.08 ± 0.24 | 0.69 |

P value; comparison between post-induction hypotension and 2 minutes after phenylephrine
